# Quality of Life in Patients With Scabies

**DOI:** 10.7759/cureus.98285

**Published:** 2025-12-02

**Authors:** Sadaf Malik, Azra Jabin, Sanan Khan, Muhammad Junaid Khan, Filza Ajmal, Areeba Khan, Syed Ahmad Shah

**Affiliations:** 1 Dermatology, Lady Reading Hospital-Medical Teaching Institution (MTI), Peshawar, PAK; 2 Dermatology, Hayatabad Medical Complex, Peshawar, PAK; 3 General Medicine, Lady Reading Hospital-Medical Teaching Institution (MTI), Peshawar, PAK

**Keywords:** dermatology life quality index, dermatology quality of life index, diagnosis of scabies, neglected tropical disease, neglected tropical disease, psychological well-being, psychosocial impact, quality of life, scabies, scabies treatment protocol

## Abstract

Scabies is a common parasitic skin infection that causes significant morbidity worldwide, particularly in resource-limited settings like Pakistan. Despite its prevalence, the psychosocial and quality of life (QoL) impact of scabies is often under-recognized in routine clinical care. This study aimed to determine the impact of scabies on the QoL in patients presenting to a tertiary care hospital in Peshawar. A cross-sectional study was conducted at the Department of Dermatology, Postgraduate Medical Institute (PGMI)/Lady Reading Hospital, Peshawar, between August 2023 and February 2024. A total of 164 patients aged 15-50 years with clinically diagnosed scabies were enrolled. QoL was assessed using the Modified Dermatology Life Quality Index (mDLQI), and scores were categorized into no, mild, moderate, or severe effect. Of 164 patients, 26 (15.9%) reported no effect on QoL, 35 (21.3%) mild, 54 (32.9%) moderate, and 49 (29.9%) severe effect. Overall, 62.8% experienced moderate to severe impairment. Scabies significantly affects patients’ QoL, and routine QoL assessment should be incorporated into dermatologic management to address both physical and psychosocial aspects of the disease.

## Introduction

Scabies is a contagious parasitic skin infestation caused by *Sarcoptes scabiei* var. hominis, affecting an estimated 200-300 million people globally each year [1]. It is recognized by the World Health Organization as a neglected tropical disease due to its high prevalence, particularly in low- and middle-income countries [2]. Families living in small closed spaces or those living in refugee or internally displaced person camps face an especially high risk because overcrowded conditions and close interaction among diverse communities create an environment that promotes rapid mite transmission [2,3].

Although scabies is generally considered a mild and non-fatal infection, it can cause significant morbidity and substantial disruption in patient quality of life (QoL) by its characteristics intense pruritus and a papular or vesicular rash, often disrupting sleep and leading to fatigue, psychological distress, and social stigma, leading to impaired QoL [3,4]. Despite this significant burden, clinical management typically focuses on treating the infection, while the psychosocial impact remains overlooked [5].

Previous studies from India, Turkey, and the Solomon Islands have shown variability in the QoL impact, reflecting differences in demographics, health access, and cultural perceptions [6-8]. However, there is a paucity of local data from Pakistan. This study aimed to evaluate the impact of scabies on the QoL among patients presenting to a tertiary care dermatology department in Peshawar.

## Materials and methods

Operational definitions

Scabies

A patient was labelled to have scabies if he/she has symptoms of intense itching and pimples-like rash along with microscopic scabies mite burrows into the upper layer of the skin, diagnosed by the consultant dermatologist, based on “The 2020 International Alliance for the Control of Scabies (IACS) consensus criteria for the diagnosis of scabies” [9].

Quality of Life

It was assessed using the Modified Dermatology Life Quality Index (mDLQI) score [10], which evaluates the impact of scabies on patients’ QoL. The effect was categorized as follows: no effect for scores 0-1, mild effect for scores 2-5, moderate effect for scores 6-10, and severe effect for scores of 11 or more.

Study duration

Sampling collection was done from August 5, 2023, to February 5, 2024.

Study design and setting

It is a cross-sectional study done in the Department of Dermatology, Lady Reading Hospital-Medical Teaching Institution (MTI), Peshawar.

Sampling technique

This study uses a non-probability consecutive sampling technique.

Sample size

Sample size was calculated using the World Health Organization sample size calculator software with a confidence level (1-α) of 95%, absolute precision (d) of 5%, and an expected frequency of no effect on QoL in patients with scabies (P) = 12.1% [11]. Based on these parameters, the calculated sample size (n) was 164.

Sample selection

The inclusion criteria for sample collection included patients of all genders, aged 14 to 50 years, who presented with scabies infection. Meanwhile, those patients with a documented history of other skin conditions apart from scabies, previous history of depression, and current or previous use of any antidepressants, as well as antipsychotics, pregnant women, and patients with chronic kidney and chronic liver diseases were excluded from the sample.

Data collection technique

After approval of the study proposal by the Ethical Committee, informed consent was obtained from all participants. Patients who fulfilled the inclusion criteria were selected, and the mDLQI was administered to each patient through a formal interview to assess the impact of scabies on QoL (as per the operational definition). The questionnaire [10] was used under license ID: CUQoL4074 from Cardiff University School of Medicine. All patients were prescribed appropriate treatment for scabies by the consultant dermatologist, and strict confidentiality and anonymity of participants were maintained throughout the study.

Data analysis

Data analysis was performed using SPSS version 29.0 (IBM Corp., Armonk, NY, US). The numeric variables (age, DLQI score) were presented as mean ± standard deviation (SD), while frequency and percentages were used for categorical variables (gender, site of disease, and QoL grade). QoL grade was stratified by age, gender, and site of disease to deal with effect modifiers. A post-stratification Pearson chi-squared test was used by keeping 5% level of significance.

Ethical considerations

Ethical approval was obtained from the institutional review board via letter Reference No. 658.1/LRH/MTI dated July 27, 2023. All patients' participation was voluntary and anonymous.

## Results

This study included 164 patients with clinically diagnosed scabies. The mean age was 32.41 ± 10.92 years, the mean disease duration was 23.23 ± 4.43 days, and the mean mDLQI score was 7.20 ± 4.21. Among patients, 88 (53.7%) were men and 76 (46.3%) were women. The mean duration of illness in our study was 23.23 days (Table 1).

**Table 1:** Demographics and Summary Statistics (N = 164) N: sample size (total); n: number of cases in each category; %: percentage of cases in each category; ±SD: standard deviation; mDLQI: Modified Dermatology Life Quality Index

Variable	Value
Male, n (%)	88 (53.7%)
Females, n (%)	76 (46.3%)
Age (years), mean ± SD	32.41 ± 10.92
Duration of disease (days), mean ± SD	23.23 ± 4.43
mDLQI score, mean ± SD	7.20 ± 4.21

The distribution of mDLQI categories and stratifications by age, gender, and the site of involvement are shown in Tables 2-5. The majority of the patients (62.8%) experienced moderate to severe QoL impairment (score ≥ 6), while 15.9% patients reported no effect on QoL, and 21.3% patients reported a mild effect on QoL. Commonly reported issues included disturbed sleep, embarrassment, difficulty in social interactions, and emotional distress. The Pearson chi-squared test was used to determine statistical significance, with a p-value of <0.05 considered significant. There was no significant difference between the genders, age, and site of infection on QoL (p > 0.05), though most patients with genital and back involvement reported a severe effect on QoL than other parts of the body involved (Table 5).

**Table 2:** Quality of Life (mDLQI)–Frequency Distribution (n = 164) mDLQI: Modified Dermatology Life Quality Index; N: sample size (total); n: number of cases in each category; %: percentage of cases in each category

mDLQI category	Frequency (n)	Percent (%)
No effect (0–1)	26	15.9
Mild effect (2 - 5)	35	21.3
Moderate effect (6-10)	54	32.9
Severe effect (≥11)	49	29.9
Total (N)	164	100

**Table 3:** Stratification of Quality of Life, Stratified by Age Group The association between age group and mDLQI category was assessed using the Pearson chi-squared test (χ²). A p-value < 0.05 was considered statistically significant. The result was not statistically significant χ²(df = 6, N = 164) = 8.3, p = 0.21). χ²: chi-squared test = 8.3 mDLQI: Modified Dermatology Life Quality Index; N: sample size (total); df: degrees of freedom

mDLQI category	14-30 years	31-40 years	41-50 years	Total	p-value (chi-squared)
No effect	13	4	9	26	
Mild effect	14	9	12	35	
Moderate effect	25	12	17	54	
Severe effect	20	20	9	49	
Total	72	45	47	164	χ²(6, N = 164) = 8.3, p = 0.21

**Table 4:** Stratification of Quality of Life by Gender The association between gender and mDLQI category was assessed using the Pearson chi-squared test (χ²). A p-value < 0.05 was considered statistically significant. The result was not statistically significant, χ²(3, N = 164) = 0.25, p = 0.97, indicating no significant relationship between gender and mDLQI category. mDLQI: Modified Dermatology Life Quality Index; n: number of cases in each category; %: percentage of cases in each category; N: sample size (total)

mDLQI categories	Male n (%)	Female n (%)	Total	p-value (chi-squared)
No effect	15 (57.7%)	11 (42.3%)	26	
Mild effect	18 (51.4%)	17 (48.6%)	35	
Moderate effect	29 (53.7%)	25 (46.3%)	54	
Severe effect	26 (53.1%)	23 (46.9%)	49	
Total	88 (53.7%)	76 (46.3%)	164	χ²(3, N = 164) = 0.25, p = 0.97

**Table 5:** Stratification of Quality of Life by the Site of Disease The association between the lesion site and mDLQI category was assessed using the Pearson chi-squared test (χ²). A p-value < 0.05 was considered statistically significant. The result was not statistically significant, χ²(12, N = 164) = 13.91, p = 0.31, indicating no significant relationship between the lesion site and mDLQI category. mDLQI: Modified Dermatology Life Quality Index; n: number of cases in each category; %: percentage of cases in each category; N: sample size (total)

mDLQI category	Face n (%)	Chest n (%)	Back n (%)	Limbs n (%)	Genitalia n (%)	Total	p-value (chi-squared)
No	8 (30.8%)	7 (26.9%)	4 (15.4%)	4 (15.4%)	3 (11.5%)	26	
Mild	10 (28.6%)	8 (22.9%)	3 (8.6%)	9 (25.7%)	5 (14.3)	35	
Moderate	12 (22.2%)	13 (24.1%)	10 (18.5%)	11 (20.4%)	8 (14.8%)	54	
Severe	7 (14.3%)	7 (14.3%)	16 (32.7%)	8 (16.3%)	11 (22.4%)	49	
Total	37	35	33	32	27	164	χ²(12, N = 164) = 13.91, p = 0.31

## Discussion

This study demonstrates that scabies has a significant negative impact on QoL, with nearly two-thirds of patients experiencing moderate to severe impairment with a mean mDLQI of 7.20 ± 4.21. These findings are consistent with studies from Turkey and India, which reported similar QoL impact patterns [6,7]. In contrast, a study from the Solomon Islands reported higher rates of no or mild impact, likely due to earlier detection and better community awareness [8], while Paudel et al. reported a higher mean DLQI (12.9 ± 5.9), indicating a very large effect. Differences may reflect disease duration, socioeconomic context, and access to care [5]. Koç Yıldırım et al. found that 72.2% of patients experienced moderate to severe impairment in QoL, correlating positively with depression and anxiety. In our study, 62.8% of patients experienced moderate to severe impairment, which is slightly lower but still substantial. While our study did not explore psychological factors such as depression or anxiety, the high rate of QoL impairment aligns with their findings, indicating that scabies can severely affect patients' well-being [12]. Jackson. et al. reported 72% of the patients have disturbed sleep, mostly because of itching [13]; our study closely followed this result as well.

Scabies affects QoL not only through physical symptoms such as pruritus but also via psychological and social consequences including stigma, embarrassment, and disrupted daily functioning [14,15]. Addressing these factors requires more than pharmacological intervention; it requires integrated care, patient education, and community-level awareness interventions [13-17].

Clinically, our results reinforce the need for dermatologists and public health practitioners to incorporate patient-centered outcomes such as DLQI when evaluating scabies. A moderate to severe QoL impairment is common and may warrant adjunctive therapeutic strategies (e.g., care bundles, symptomatic itch management, sleep hygiene advice, and psychosocial support) beyond mite eradication to help in the pruritus and stigmatization management as in other chronic dermatological disease management [18]. Treatment of household contacts, education on transmission, and early diagnosis may also reduce the duration of symptoms and the downstream QoL impact. Future research should include longitudinal follow-up to assess changes in DLQI with treatment, include formal screening for psychiatric comorbidity, and explore the effect of sociodemographic variables (e.g., education, income, and overcrowding) and lesion chronicity.

The limitation of this study is that this was a single-center study with a hospital-based sample, which may not fully represent the community prevalence. Additionally, only descriptive analysis was performed; future studies should explore predictive factors of QoL impairment.

## Conclusions

Scabies significantly impairs the QoL in affected patients, especially in resource-limited settings. Routine QoL assessment, patient counseling, and community-based interventions should be integral components of scabies control programs. The physical discomfort from persistent itching, coupled with emotional distress and sleep disturbance, highlights that scabies is not merely a contagious skin infestation but a condition with broad psychosocial implications. These findings emphasize the need for clinicians to recognize the wider burden of scabies and to assess patient well-being as part of comprehensive disease management.

An integrated care approach is essential to effectively address the multifaceted impact of scabies. In addition to pharmacological treatment, interventions should focus on symptom control, sleep hygiene, psychological support, and education to reduce stigma and transmission within the community. Incorporating quality-of-life assessments into clinical practice can guide targeted support, improve treatment outcomes, and ultimately enhance patients’ overall well-being.
